# Macroalgae Extracts From Antarctica Have Antimicrobial and Anticancer Potential

**DOI:** 10.3389/fmicb.2018.00412

**Published:** 2018-03-08

**Authors:** Rosiane M. Martins, Fernanda Nedel, Victoria B. S. Guimarães, Adriana F. da Silva, Pio Colepicolo, Claudio M. P. de Pereira, Rafael G. Lund

**Affiliations:** ^1^Post-Graduate Program in Biochemistry and Bioprospecting, Federal University of Pelotas, Pelotas, Brazil; ^2^Post-Graduate Program in Dentistry, School of Dentistry, Federal University of Pelotas, Pelotas, Brazil; ^3^Department of Biochemistry, Institute of Chemistry, State University of São Paulo, São Paulo, Brazil; ^4^Center of Chemical, Pharmaceutical and Food Sciences, Federal University of Pelotas, Pelotas, Brazil

**Keywords:** marine natural products, seaweeds, antibacterial agents, antifungal agents, antineoplastic agents

## Abstract

**Background:** Macroalgae are sources of bioactive compounds due to the large number of secondary metabolites they synthesize. The Antarctica region is characterized by extreme weather conditions and abundant aggregations of macroalgae. However, current knowledge on their biodiversity and their potential for bio-prospecting is still fledging. This study evaluates the antimicrobial and cytotoxic activity of different extracts of four macroalgae (*Cystosphaera jacquinotii, Iridaea cordata, Himantothallus grandifolius*, and *Pyropia endiviifolia*) from the Antarctic region against cancer and non-cancer cell lines.

**Methods:** The antimicrobial activity of macroalgae was evaluated by the broth microdilution method. Extracts were assessed against *Staphylococcus aureus* ATCC 19095, *Enterococcus faecalis* ATCC 4083, *Escherichia coli* ATCC29214, *Pseudomonas aeruginosa* ATCC 9027, *Candida albicans* ATCC 62342, and the clinical isolates from the human oral cavity, namely, *C. albicans* (3), *C. parapsilosis, C. glabrata, C. lipolytica*, and *C. famata*. Cytotoxicity against human epidermoid carcinoma (A-431) and mouse embryonic fibroblast (NIH/3T3) cell lines was evaluated with MTT colorimetric assay.

**Results:** An ethyl acetate extract of *H. grandifolius* showed noticeable antifungal activity against all fungal strains tested, including fluconazole-resistant samples. Cytotoxicity investigation with a cancer cell line revealed that the ethyl acetate extract of *I. cordata* was highly cytotoxic against A-431 cancer cell line, increasing the inhibitory ratio to 91.1 and 95.6% after 24 and 48 h exposure, respectively, for a concentration of 500 μg mL^−1^. Most of the algal extracts tested showed little or no cytotoxicity against fibroblasts.

**Conclusion:** Data suggest that macroalgae extracts from Antarctica may represent a source of therapeutic agents.

**HIGHLIGHTS**

- Different macroalgae samples from Antarctica were collected and the lyophilized biomass of each macroalgae was extracted sequentially with different solvents
- The antimicrobial and anticancer potential of macroalgae extracts were evaluated
- Ethyl acetate extract of *H. grandifolius* showed noticeable antifungal activity against all the fungal strains tested, including fluconazole-resistant samples
- Ethyl acetate extract of *I. cordata* was highly cytotoxic against the A-431 cancer cell line
- Most of the algal extracts tested showed little or no cytotoxicity against normal cell lines

## Introduction

Nature is an amazing source of chemical diversity and naturally derived compounds have unique pharmacological properties. A review of natural products (NPs) over a period of the 34 years, from 1981 to 2014, revealed that ~40% of the developed therapeutic agents approved by the FDA were NPs, their derivatives, or synthetic mimetic products related to NPs (Newman and Cragg, 2016). As a consequence of the increasing demand for natural products that may be used in new therapeutic drugs, interest in marine organisms has increased. Although terrestrial life is extraordinarily diverse, a greater biodiversity is found in the world's oceans, represented by 34 of the 36 phyla (Chakraborty et al., 2009). The oceans cover more than 70% of the earth's surface and contain an extraordinary diversity of life, with an enormous resource for potential therapeutic agents (Ngo et al., 2012).

Accordingly, products of marine origin have been increasingly used in human health, a trend that is likely to continue in the future. Due to the harsh conditions which marine organisms have to cope with, such as low temperatures, low light availability, and high pressure, marine organisms respond by synthesizing a number of secondary metabolites, some of which have potent pharmacological properties (Villa and Gerwick, 2010; Mayer et al., 2013; Gribble, 2015).

Algae are a heterogeneous group of marine organisms commonly divided into microalgae and macroalgae, based on size. Macroalgae, also known as seaweeds, have a complex and dynamic taxonomy. The three main algal phyla are Rhodophyta (red algae), Ochrophyta (brown algae), and Chlorophyta (green algae) (Leal et al., 2013).

Macroalgae are known to produce molecules with great chemical diversity that have relevant effects on human health. There are numerous reports of compounds derived from macroalgae with a broad range of biological effects, such as antibacterial, antifungal, antiviral, antitumoral, anticoagulant, and antioxidant activities (El Gamal, 2010; Shalaby, 2011; Deveau et al., 2016; Pinteus et al., 2016; Vijayan et al., 2016). Compounds with biological activities have been detected in green, brown and red algae. Thus, there is a growing interest in researching the positive effect of algae on human health.

The Antarctic region is characterized by an extremely abundant aggregation of macroalgae, strongly contrasting with its lack of a diversified terrestrial flora (Wiencke et al., 2007). Although considerable progress has been made in recent years, our knowledge on the region's macroalgae is still fragmented. Recent studies on these algae have revealed a fatty acid profile (Santos et al., 2017) and steroids (Pereira et al., 2017). Moreover, other studies have been carried out in the Chilean sub-Antarctic region where these algae are abundant and may be exploited for bioprospecting research (Wiencke et al., 2007; Astorga-España and Mansilla, 2014; Fujii et al., 2014; Kim et al., 2016). To date, there are few published reports on pharmacological investigation of seaweeds from Antarctica. Current study evaluates seaweed extracts from Antarctica to determine their antimicrobial potential and cytotoxic activity against cancer and non-cancer cells.

## Materials and methods

### Macroalgae samples

**Figure 1:**
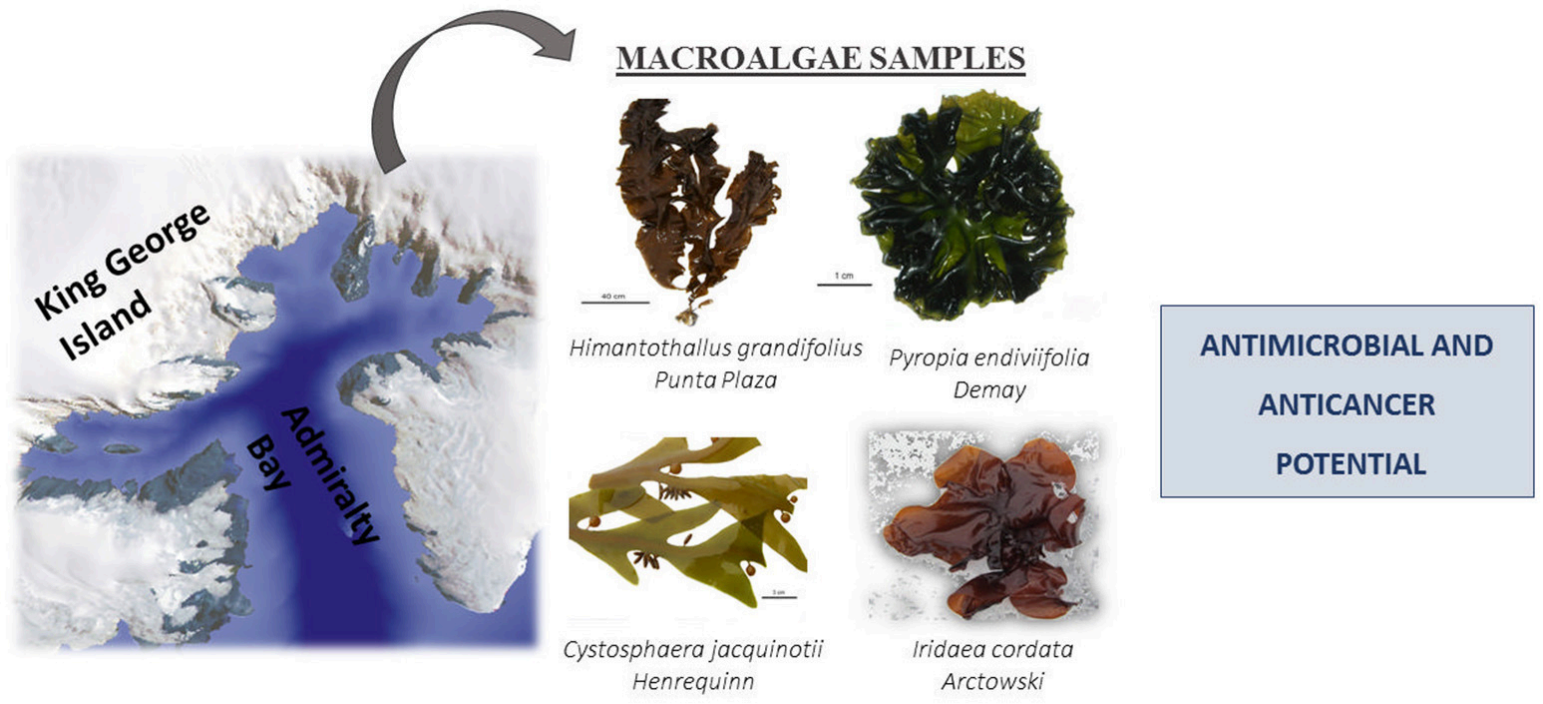
Macroalgae species assessed in this study.

Samples of macroalgae *C. jacquinotii, I. cordata, H. grandifolius*, and *P. endiviifolia* were collected during the expeditions integrating the Brazilian Antarctic Program, between January and February 2012, in Admiralty Bay, at different sites on King George Island, Antarctica. Collected macroalgae were dried at room temperature and placed in plastic bags to protect them from light Figure 1. The project was supported by the Botanical Institute of São Paulo (Government of the state of São Paulo, Brazil). Dr. Toyota Fujii Mutue, an expert on phycology, identified the authenticity of the collected algae biomass and the material was compared to a database of Botanic Institute. Part of this collection was recently reported in the literature by Fujii et al. (2014).

At each sampling site, macroalgae were randomly collected from the intertidal region by a destructive method inside 1 m^2^ quadrants along three transects of 100 m, parallel to the coast, during low tide. Temperature, salinity, and pH were monitored using a double channel multiprobe (HQ40D Hexis/Hach—Loveland CO USA) on three surface water samples for each sampling point (Pellizzari et al., 2017). Table 1 provides information on the macroalgae collected and used in this study. The seaweeds were washed with local seawater to remove all extraneous matter, such as epiphytes, sand particles, pebbles and shells, and transported to the laboratory in plastic bags.

**Table 1:** Collection information and species used in the experiments.

**Species**	**Collection data**
*Cystosphaera jacquinotii* (Montagne) Skottsberg	Punta Henrequinn (62°7′32.77″ × 58°23′38.03″) 16th January, 2012
*Iridaea cordata* (Turner) Bory de Saint-Vincent	Arctowski (62°7′32.77″ × 58°23′38.03″) 3rd February, 2012
*Himantothallus grandifolius* (A.Gepp & E.S.Gepp) Zinova	Punta Plaza (62°5′34.82″ × 58°24′15.27″) 29th January, 2012
*Pyropia endiviifolia* (A.Gepp & E.Gepp) H.G.Choi & M.S.Hwang	Demay (62°13′21.01″ × 58°26′36.59″) 27th January, 2012

### Preparation of extracts

The lyophilized biomass (5 g) of each macroalga was extracted sequentially with 150 mL of different solvents (i.e., hexane/chloroform/ethyl acetate/ethanol) using a Soxhlet apparatus for 6 h, at a temperature not exceeding the boiling point of the solvent. Procedure was repeated 2–3 times to obtain a sizable quantity of extract. The solvent was removed from each extract under reduced pressure, at 20–25°C. They were finally dried under a N_2_ stream. Extracts were weighed and stored at −20°C until use. Yields of the extracts ranged between 1 and 2%.

### Antimicrobial activity

#### Microorganisms

The antimicrobial activity of algal extracts [hexane (HE), chloroform (CE), ethyl acetate (EA), and ethanol (EE) extracts] was determined by two representative Gram-positive bacteria (*Staphylococcus aureus* ATCC19095 and *Enterococcus faecalis* ATCC4083), two Gram-negative bacteria (*Escherichia coli* ATCC29214 and *Pseudomonas aeruginosa* ATCC9027), and eight strains, representing five different species of yeasts (*Candida albicans* ATCC62342 and clinical isolates of *C. albicans* from the human oral cavity (3), *C. parapsilosis, C. glabrata, C. lipolytica*, and *C. famata*). Current assay was approved by the institution's Committee for Ethics in Research (Protocol n. 033/2006). The bacteria and fungi used in this study were selected due to their medical importance in infectious diseases. Target strains were maintained in Mueller-Hinton agar and Sabouraud dextrose agar, respectively, at 4°C and reactivated before use.

#### The broth microdilution test

The antimicrobial assay for yeast (M27-A3) and for bacteria (M7-A7) was carried with broth micro-dilution method according to guidelines of the Clinical and Laboratory Standards Institute (CLSI) (Clinical and Laboratory Standards Institute, 2006, 2008).

Microbial strains were primarily inoculated for overnight growth. Several colonies were directly suspended in a saline solution until turbidity was reached, equivalent to a McFarland standard of 0.5. The suspension was then adjusted to obtain a working concentration of 5 × 10^5^ colony forming units (CFUs) mL^−1^. Mueller-Hinton broth (BD, Sparks MD USA) and RPMI-1640 (Sigma, St Louis MO USA) buffered to pH 7.0 with MOPS were used for growing and diluting the bacterial and fungal suspensions, respectively. Dilutions of tetracycline, chloramphenicol, and fluconazole were used as reference compounds to compare data between independent experiments and as indicators for assessing the relative level of inhibition of the samples tested. Dimethyl sulfoxide (DMSO) was the diluent to obtain the desired concentration of the extracts and reference drugs.

The diluted samples were serially transferred, in duplicate, to 96-well microplates, and 100 μL of microbial inoculum were added to achieve a final volume of 200 μL. The concentrations ranged between 1 and 500 μg mL^−1^. Final concentration of DMSO in the assay did not exceed 0.5%, which is nontoxic to cells (Ritter et al., 2015). Control experiments to test microbial viability and sterility of the medium and extract were also carried out. The plates were incubated at 37°C for 24 h for bacteria and 48 h for fungi. The plates were read before and after incubation at 630 nm for bacteria and 590 nm for fungi. The antimicrobial effect was characterized by IC_50_, which is the concentration that induces 50% inhibition of bacterial/fungal growth relative to the growth control, and by MIC, which is the lowest concentration of the substance required for complete inhibition of bacterial and fungal growth after incubation time. IC_50_ rates were determined by logarithmic graphs of growth inhibition vs. concentration. Tests were carried out in duplicate.

#### Determination of MMC

After determining MIC, the minimal microbicidal concentration (MMC) was measured. Aliquots of 20 μL from the wells were plated in Mueller Hinton agar (for bacteria) or in Sabouraud dextrose agar (for fungi) and incubated at 37°C for 24 h. MMC is defined as the lowest concentration of each extract that resulted in no cell growth on the plate surface.

### Determination of cytotoxicity

#### Cell culture

The cytotoxicity was evaluated by human epidermoid carcinoma (A-431) and mouse embryonic fibroblast (NIH/3T3) cell lines. Cells were obtained from the Rio de Janeiro Cell Bank (PABCAM, Federal University of Rio de Janeiro, Brazil) and cultured in Dulbecco's modified Eagle's medium, supplemented with 10% fetal bovine serum (FBS) purchased from Cultilab (Campinas, Brazil) and Gibco (Grand Island NY USA), respectively. The cells were grown at 37°C in an atmosphere of 95% humidified air and 5% CO_2_. Experiments were performed with cells in the logarithmic phase of growth.

#### The MTT assay

Cell viability was determined by measuring the reduction of soluble MTT [3-(4,5-dimethylthiazol-2-yl)-2,5-diphenyltetrazolium bromide] to water insoluble formazan (Henn et al., 2011). Briefly, cells were seeded at a density of 2 × 10^4^ cells per well, in 96-well plates, in a volume of 100 μL and grown at 37°C in a humidified atmosphere of 5% CO_2_/95% air for 24 h before being used in the MTT assay. The cells were incubated with different concentrations of extracts (31.25–500 μg mL^−1^) for 24 and 48 h. These compounds were dissolved in DMSO and added to DMEM, supplemented with 10% FBS, to the desired concentrations. The final DMSO concentration in the culture medium never exceeded 0.5%, and a control group exposed to an equivalent concentration of DMSO was evaluated. After incubation, the media were removed, and 180 μL of DMEM and 20 μL of MTT (5 mg MTT/mL solution) were added to each well. Plates were incubated for an additional 3 h period, and the medium was discarded. DMSO (200 μL) was added to each well, and the formazan was solubilized on a shaker, for 5 min, at 100 × g. The absorbance of each well was read on a microplate reader (MR-96A, Mindray Shenzhen, China) at a wavelength of 492 nm. The percent inhibition of cellular growth was determined as follows: inhibitory rate = (1- Abs_492treated cells_/Abs_492control cells_) × 100. All observations were validated by at least two independent experiments.

## Results and discussion

### Antimicrobial activity

Table 2 shows the results of antimicrobial screening of the extracts and drug standards (characterized by IC_50_, MIC, and MMC rates, μg mL^−1^) against a panel of selected microbial strains. EA of *C. jacquinotii* proved to have the highest antibacterial activity among the different extracts, with IC_50_ = 46.75 μg mL^−1^ and MIC = 500 μg mL^−1^ against *E. faecalis*. None of the other extracts showed *in vitro* antibacterial activity against the tested bacterial strains at concentrations up to 500 μg mL^−1^.

**Table 2:** The *in vitro* antimicrobial activities of extracts and reference drugs against human pathogens.

**Species (Extract)**	**Assay**	**Microbial strains**											
	**(μg mL^−1^)**	***C.a***	***C.a*1**	***C.a*2**	***C.a*3**	***C.p***	***C.g***	***C.l***	***C.f***	***S.a***	***E.c***	***P.a***	***E.f***
*Cystosphaera jacquinotii* (HE)	IC_50_	–	–	–	–	–	–	–	–	–	–	–	–
	MIC	–	–	–	–	–	–	–	–	–	–	–	–
	MMC	nd	nd	nd	nd	nd	nd	nd	nd	nd	nd	nd	nd
*Cystosphaera jacquinotii* (CE)	IC_50_	–	–	–	–	–	–	–	–	–	–	–	–
	MIC	–	–	–	–	–	–	–	–	–	–	–	–
	MMC	nd	nd	nd	nd	nd	nd	nd	nd	nd	nd	nd	nd
*Cystosphaera jacquinotii* (AE)	IC_50_	–	–	–	–	174.4	–	–	–	–	–	–	46.75
	MIC	–	–	–	–	500	–	–	–	–	–	–	500
	MMC	nd	nd	nd	nd	500	nd	nd	nd	nd	nd	nd	–
*Cystosphaera jacquinotii* (EE)	IC_50_	–	–	–	–	–	–	–	–	–	–	–	–
	MIC	–	–	–	–	–	–	–	–	–	–	–	–
	MMC	nd	nd	nd	nd	nd	nd	nd	nd	nd	nd	nd	nd
*Iridea cordata* (HE)	IC_50_	–	–	–	–	–	–	–	–	–	–	–	–
	MIC	–	–	–	–	–	–	–	–	–	–	–	–
	MMC	nd	nd	nd	nd	nd	nd	nd	nd	nd	nd	nd	nd
*Iridea cordata* (CE)	IC_50_	–	–	–	–	–	–	–	–	–	–	–	–
	MIC	–	–	–	–	–	–	–	–	–	–	–	–
	MMC	nd	nd	nd	nd	nd	nd	nd	nd	nd	nd	nd	nd
*Iridea cordata* (AE)	IC_50_	–	–	–	–	–	–	–	–	–	–	–	–
	MIC	–	–	–	–	–	–	–	–	–	–	–	–
	MMC	nd	nd	nd	nd	nd	nd	nd	nd	nd	nd	nd	nd
*Iridea cordata* (EE)	IC_50_	–	–	–	–	–	–	–	–	–	–	–	–
	MIC	–	–	–	–	–	–	–	–	–	–	–	–
	MMC	nd	nd	nd	nd	nd	nd	nd	nd	nd	nd	nd	nd
*Himantothallus grandifolius* (HE)	IC_50_	–	–	–	–	–	–	–	–	–	–	–	–
	MIC	–	–	–	–	–	–	–	–	–	–	–	–
	MMC	nd	nd	nd	nd	nd	nd	nd	nd	nd	nd	nd	nd
*Himantothallus grandifolius* (CE)	IC_50_	–	–	–	–	–	–	–	–	–	–	–	–
	MIC	–	–	–	–	–	–	–	–	–	–	–	–
	MMC	nd	nd	nd	nd	nd	nd	nd	nd	nd	nd	nd	nd
*Himantothallus grandifolius* (EA)	IC_50_	49.59	46.75	17.40	4.94	19.68	29.96	13.63	25.84	–	–	–	–
	MIC	500	250	62.5	31.25	250	125	62.5	125	–	–	–	–
	MMC	500	250	62.5	31.25	250	125	62.5	125	nd	nd	nd	nd
*Himantothallus grandifolius* (EE)	IC_50_	334.40	172.60	101.60	39.30	136.30	295.51	269.60	29.96	–	–	–	–
	MIC	–	–	–	500	–	–	–	125	–	–	–	–
	MMC	nd	nd	nd	500	nd	nd	nd	125	nd	nd	nd	nd
*Pyropia endiviifolia* (HE)	IC_50_	–	–	–	–	–	–	–	–	–	–	–	–
	MIC	–	–	–	–	–	–	–	–	–	–	–	–
	MMC	nd	nd	nd	nd	nd	nd	nd	nd	nd	nd	nd	nd
*Pyropia endiviifolia* (CE)	IC_50_	–	–	–	–	–	–	–	–	–	–	–	–
	MIC	–	–	–	–	–	–	–	–	–	–	–	–
	MMC	nd	nd	nd	nd	nd	nd	nd	nd	nd	nd	nd	nd
*Pyropia endiviifolia* (EA)	IC_50_	–	402.91	–	–	–	–	–	–	–	–	–	–
	MIC	–	–	–	–	–	–	–	–	–	–	–	–
	MMC	nd	nd	nd	nd	nd	nd	nd	nd	nd	nd	nd	nd
*Pyropia endiviifolia* (EE)	IC_50_	–	–	–	–	–	–	–	–	–	–	–	–
	MIC	–	–	–	–	–	–	–	–	–	–	–	–
	MMC	nd	nd	nd	nd	nd	nd	nd	nd	nd	nd	nd	nd
**REFERENCE DRUGS**													
Fluconazole	IC_50_	5.34	21.21	<1	<1	<1	14,58	<1	<1	nt	nt	nt	nt
	MIC	–	–	1.95	31.25	3.9	125	3.9	3.9	nt	nt	nt	nt
	MMC	nd	nd	62.5	31.25	7.8	250	7.8	3.9	nt	nt	nt	nt
Chloramphenicol	IC_50_	nt	nt	nt	nt	nt	nt	nt	nt	2.1	148.7	1.92	1.94
	MIC	nt	nt	nt	nt	nt	nt	nt	nt	3.9	250	3.9	3.9
	MMC	nt	nt	nt	nt	nt	nt	nt	nt	62.5	500	31.25	31.25
Tetracycline	IC_50_	nt	nt	nt	nt	nt	nt	nt	nt	<1	36.39	<1	<1
	MIC	nt	nt	nt	nt	nt	nt	nt	nt	<1	62.5	<1	<1
	MMC	nt	nt	nt	nt	nt	nt	nt	nt	3.9	500	3.9	15.62

*(–), >500 μg mL^−1^; nd, not determined, as the MIC was >500 μg mL^−1^; nt, not tested; HE, Hexane extract; CE, chloroform extract; EA, ethyl acetate extract; EE, ethanol extract*;

*Microbial strains: Ca, Candida albicans ATCC62342; Ca1, Candida albicans clinical isolate 1; Ca2, Candida albicans clinical isolate 2; Ca3, Candida albicans clinical isolate 3; Cp, Candida parapsilosis clinical isolate; Cg, Candida glabrata clinical isolate; Cl, Candida lipolytica clinical isolate; Cf, Candida famata clinical isolate; Sa, Staphylococcus aureus ATCC19095; Ec, Escherichia coli ATCC29214; Pa, Pseudomonas aeruginosa ATCC9027; Ef, Enterococcus faecalis ATCC4083*.

Further, EA of *H. grandifolius* exhibited the most notable antifungal activity of all the extracts studied. In addition, it showed not only fungistatic but also fungicidal activity. The ethyl acetate extract of *H. grandifolius* showed IC_50_ rates ranging between 4.94 and 49.50 μg mL^−1^ and MIC and MMC rates ranging between 31.25 and 500 μg mL^−1^ against fungal strains. The other extracts showed only low or no activity against the fungal strains of the panel at concentrations up to 500 μg mL^−1^.

Ethyl Acetate (EA) of *H. grandifolius* was the most active against the fluconazole-resistant strains *C. albicans* ATCC62342 and *C. albicans* clinical isolate 1. Although the IC_50_ rates were lower when compared to those of fluconazole, MIC and MMC were better, with rates 500 and 250 μg mL^−1^ against *C. albicans* ATCC62342 and *C. albicans* clinical isolate 1, respectively. These rates are >500 μg mL^−1^ for fluconazole. The extract exhibited activity equal to fluconazole against *C. albicans* clinical isolate 3 (MMC 62.5 μg mL^−1^) and *C. albicans* clinical isolate 4 (MIC and MMC 31.25 μg mL^−1^). Furthermore, EA had an equal MIC (125 μg mL^−1^) and better MMC (125 μg mL^−1^) rates than fluconazole (MMC 250 μg mL^−1^) against *C. glabrata*.

When the different fungal strains were compared, one could perceive that *C. albicans* clinical isolate 4 was the most susceptible and *C. albicans* ATCC62342 was the most resistant strain. Results demonstrate that the sensitivity of *Candida* to seaweed extracts varies according to the different strains and species of the genus.

The genus *Candida* is the most important opportunistic fungal pathogen (Spampinato and Leonardi, 2013). Further, increase in antifungal resistance to commercial drugs and the incidence of fungal infections cause serious public health problems (Souza et al., 2016). Therefore, there is an urgent need to develop new compounds featuring low cytotoxicity but high potency to treat mycoses.

*H. grandifolius* appears to be a particularly interesting algal species, showing activity against all the fungal strains tested, including the fluconazole-resistant samples. According to Sufian et al. (2013), plant extracts with MIC rates below 1,000 μg mL^−1^ are considered noteworthy, and isolated compounds with MICs ≤ 100 μg mL^−1^ are considered active. Therefore, based on this criterion, EA was clearly the most active extract, demonstrating equal or higher rates than fluconazole, the reference drug. Although MIC/MMC rates >69 μg mL^−1^ are considered toxic for fluconazole (Clinical and Laboratory Standards Institute, 2008), EA did not display any cytotoxic effects at concentrations up to 500 μg mL^−1^, demonstrating the capacity of selectively killing yeast cells, with little damage to normal cells.

### Cytotoxicity activity

**Figure 2:**
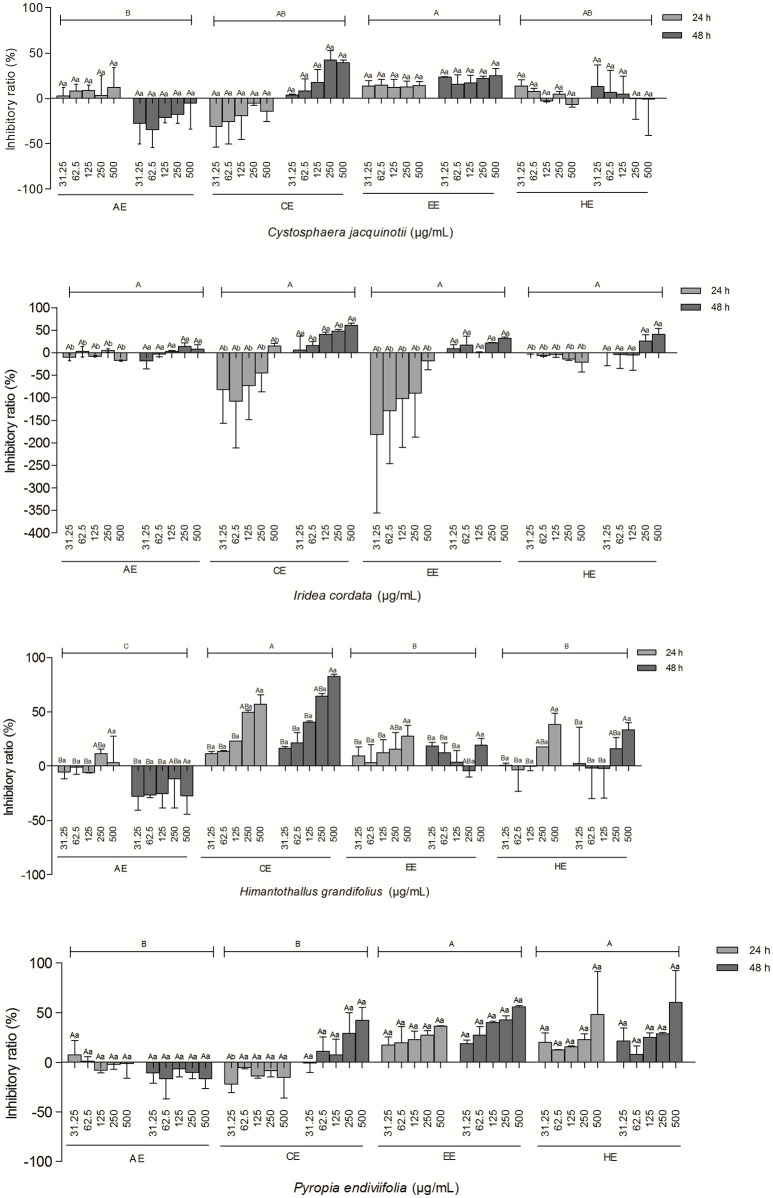
Effect of different types and concentrations of algal extracts on the inhibition of NIH/3T3 cell line following exposure for 24 and 48 h. Data are expressed as means ± SEM. Uppercase letters indicate significant differences between concentrations; lowercase letters indicate significant differences between exposure times. (
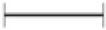
) indicates significant differences between the types of extracts. A *p*-value < 0.05 was considered significant by Tukey's test.

Results from cytotoxicity screening of extracts against NIH/3T3 cell line are shown in Figure 2. In general, the extracts presented very low or no cytotoxicity, with an inhibitory rate lower than 50%. The exception was CE of *H. grandifolius*. As shown in Figure 2, at *H. grandifolius* CE concentrations of 250 and 500 μg mL^−1^, the cytotoxicity increased to 48.3/57.1% (24/48 h) and 64.5/82.5% (24/48 h), respectively. Some extracts, such as the CE and EE of *I. cordata* were able to stimulate the growth of normal cell lines after exposure for 24 h.

Since most of the algal extracts tested showed very low or no cytotoxic effects on the normal cell line, other potentially important biological activities of these algal extracts should be explored in the future. Some extracts, such as the CE and EE of *I. cordata*, were able to stimulate the growth of the normal cell line. These data may be considered useful for other investigations, such as those exploring healing processes.

**Figure 3:**
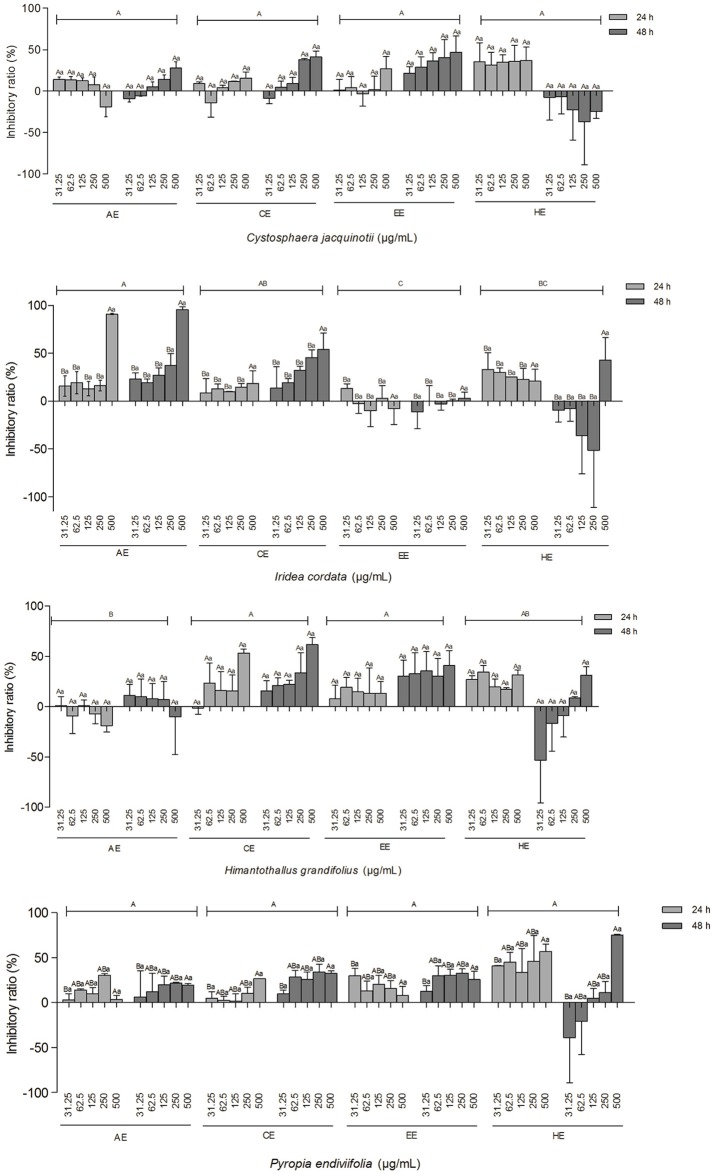
The effect of different types and concentrations of algal extracts on the inhibition of the A431 cell line following exposure for 24 and 48 h. Data are expressed as means ± SEM. Uppercase letters indicate significant differences between concentrations; lowercase letters indicate significant differences between exposure times. (
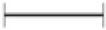
) indicates significant differences between the types of extracts. A *p*-value < 0.05 was considered significant by Tukey's test).

The cytotoxicity of the algal extracts against cancer cell line A-431 is shown in Figure 3. *C. jacquinotii* extracts had low cytotoxicity against the A-431 cell line, with an inhibitory rate lower than 50%. No significant difference was detected between different types and concentrations of algal extracts and exposure times (24 and 48 h). EA of *I. cordata* displayed the highest cytotoxic activity of all extracts tested against A-431 cells. The extract increased the inhibitory ratio to 91.1 and 95.6% after 24 and 48 h of exposure, respectively, at a concentration of 500 μg mL^−1^. There was no significant difference between A-431 cells exposed to *I. cordata* extracts for 24 and 48 h. Another study on antitumor activity of Antarctic macroalgae, including *I. cordata*, against cells from the malignant tumor Glioblastoma Multiforme was also published (Pereira et al., 2015). Another study isolated and characterized sulfated polysaccharides (SPs) from *I. cordata* and evaluated their anticancer activity against PC-3 (prostate cancer), HeLa (cervical cancer), and HT-29 (human colon adenocarcinoma). Their antitumor activities, albeit weak, proved to be interesting for medical attention and application (Kim et al., 2016). *H. grandifolius* CE and EE extracts showed the highest inhibitory activity against A-431 cells. CE concentration 500 μg mL^−1^ increased the cytotoxicity to 53.3 and 61.6% after 24 and 48 h of exposure, respectively, while the same concentration of EE increased cytotoxicity to 41% after a 48 h-exposure. However, no significant difference was detected between the two extracts. A previous study on the selective cytotoxic effect of an *H. grandifolius* hydroacetonic extract on epithelial tumor cell lines (A375, A549, Hep-2, HeLa) revealed the capacity to suppress proliferation and promote apoptosis-mediated cell death with the induction of initial stages of apoptosis in these cell lines (Gambato et al., 2014). At a concentration of 500 μg mL^−1^, the HE of *P. endiviifolia* increased the inhibitory rate to 56.6 and 74.9% after 24 and 48 h-exposure times, respectively. However, no significant differences were found among *P. endiviifolia* extracts.

Non-melanoma skin cancer is the most frequently diagnosed cutaneous malignancy, accounting for over 90% of all incidences of skin cancer (Jiang et al., 2012; Pal et al., 2013). Although skin cancer is often treated by surgery, chemotherapy or radiation therapies, the risk of recurrence and metastasis remains a concern (Jiang et al., 2012). Therefore, continuous efforts have been made to pursue and develop new approaches to treat skin cancer more effectively. In the present study, the authors detected that EA of *I. cordata* showed very strong cytotoxicity against an epidermoid carcinoma cell line (A-431) at a concentration of 500 μg mL^−1^.

### Factors associated to the bioactivity of macroalgae extracts

Several authors have suggested that the production of bioactive substances varies within a species. Variability may be due to seasonal variations, geographic distribution and growth stage or sexual maturation (Manilal et al., 2009; Patra et al., 2009; Galal et al., 2011). Our samples are brown and red, and they were collected from the Antarctic in the summer. Our results would have been different if seaweed samples had been collected during other seasons or at different growth stages and sexual maturation.

The bioactivity of the seaweed extracts may be influenced by extraction protocols and extraction capacity of the solvents used. Although a variety of solvents have been employed in screening seaweed for biological activity, it is still uncertain what type of solvent is the most effective and suitable for the extraction of compounds from seaweeds. Some studies on the effectiveness of extraction methods highlight that methanol extracts exhibit higher antimicrobial activity than ethyl acetate (Manilal et al., 2009; Galal et al., 2011). Other studies report that ethyl acetate and chloroform are better than ethanol and methanol (Patra et al., 2009). According to our antimicrobial and anti-cancer experimental results, ethyl acetate yielded better results than hexane, chloroform and ethanol. Result could be related to the presence of bioactive metabolites that are soluble in ethyl acetate but have no or little solubility in other solvents used.

Current study was limited by the relatively small yield obtained from macroalgae which, for instance, impaired testing the antimicrobial effects of the samples' algal parts. This could affect the interindividual/intraindividual changes of biological activities in macroalga, as the algae were only tested with 5 g of the material.

Demonstrating the bioactive profile of seaweed extracts from Antarctica, data suggest that seaweeds from this region may be promising for the isolation of substances with important biological potential. Fractionation or isolation of the individual components of extracts that showed antimicrobial and antitumor activities is necessary to understand the activity of each individual component and the contribution of each component to the overall activity of the extract. Further research should be undertaken to explore the bioactive compounds of the studied seaweeds to ensure their successful application as potential therapeutic tools.

## Conclusion

Current study identified the potential use of two extracts as components of future drugs. Whereas, ethyl acetate extract of *H. grandifolius* was the most active extract against fungal samples, ethyl acetate extract of *I. cordata* was the most cytotoxic against the cancer cell line tested. Moreover, the algal extracts tested were generally not cytotoxic against the non-cancer cell line.

## Author contributions

RM: Contributed to antimicrobial evaluation and drafted the paper; FN and AdS: Contributed to determination of cytotoxicity and analysis of the data; VG: Contributed to drafted the paper; PC: Contributed to collection and identification of the algal species used in this study; CdP: Contributed to preparation of extracts; RL: Designed the study, supervised the laboratory work, and contributed to critical reading of the manuscript. All the authors have read the final manuscript and approved the submission.

### Conflict of interest statement

The authors declare that the research was conducted in the absence of any commercial or financial relationships that could be construed as a potential conflict of interest.
